# Effect of oral nitrate supplementation on pulmonary hemodynamics during exercise and time trial performance in normoxia and hypoxia: a randomized controlled trial

**DOI:** 10.3389/fphys.2015.00288

**Published:** 2015-10-14

**Authors:** Nicolas Bourdillon, Jui-Lin Fan, Barbara Uva, Hajo Müller, Philippe Meyer, Bengt Kayser

**Affiliations:** ^1^Faculty of Biology and Medicine, Institut des Sciences du Sport de l'Université de Lausanne, University of LausanneLausanne, Switzerland; ^2^Department of Physiology, Faculty of Biology and Medicine, University of LausanneLausanne, Switzerland; ^3^Lemanic Neuroscience Doctoral School, University of LausanneLausanne, Switzerland; ^4^Cardiology Service, University Hospital of GenevaGeneva, Switzerland

**Keywords:** altitude, hypoxia, nitrate, NO, exercise, performance

## Abstract

**Background:** Hypoxia-induced pulmonary vasoconstriction increases pulmonary arterial pressure (PAP) and may impede right heart function and exercise performance. This study examined the effects of oral nitrate supplementation on right heart function and performance during exercise in normoxia and hypoxia. We tested the hypothesis that nitrate supplementation would attenuate the increase in PAP at rest and during exercise in hypoxia, thereby improving exercise performance.

**Methods:** Twelve trained male cyclists [age: 31 ± 7 year (mean ± SD)] performed 15 km time-trial cycling (TT) and steady-state submaximal cycling (50, 100, and 150 W) in normoxia and hypoxia (11% inspired O_2_) following 3-day oral supplementation with either placebo or sodium nitrate (0.1 mmol/kg/day). We measured TT time-to-completion, muscle tissue oxygenation during TT and systolic right ventricle to right atrium pressure gradient (RV-RA gradient: index of PAP) during steady state cycling.

**Results:** During steady state exercise, hypoxia elevated RV-RA gradient (*p* > 0.05), while oral nitrate supplementation did not alter RV-RA gradient (*p* > 0.05). During 15 km TT, hypoxia lowered muscle tissue oxygenation (*p* < 0.05). Nitrate supplementation further decreased muscle tissue oxygenation during 15 km TT in hypoxia (*p* < 0.05). Hypoxia impaired time-to-completion during TT (*p* < 0.05), while no improvements were observed with nitrate supplementation in normoxia or hypoxia (*p* > 0.05).

**Conclusion:** Our findings indicate that oral nitrate supplementation does not attenuate acute hypoxic pulmonary vasoconstriction nor improve performance during time trial cycling in normoxia and hypoxia.

## Introduction

Pulmonary hypertension is one of the most striking of all physiological responses to hypoxia (West et al., 2013). It results from an increase in pulmonary vascular resistance (PVR). The mechanism of the underlying hypoxic pulmonary vasoconstriction (HPV) is complex (Sylvester et al., 2012). It is mediated by a hypoxia-induced increase in intracellular calcium concentration, depolarizing smooth muscle in the walls of pulmonary arterioles and veins PVR (Murray et al., 1990a,b; Madden et al., 1992; Swenson, 2013). Acute exposure to severe hypoxia (11% O_2_) can increase resting mean pulmonary arterial pressure (PAP) by as much as 13–23 mmHg compared to normoxic values (Motley and Cournand, 1947; Groves et al., 1987).

Elevation in PAP during hypoxic exposure impairs resting cardiac function by increasing right atrial and ventricular end-diastolic pressures, which impedes left atrial filling (Maignan et al., 2009), an effect amplified during exercise (Swenson, 2013). Hypoxic pulmonary hypertension further leads to ventilation-perfusion mismatching during exercise (Gale et al., 1985; Torre-Bueno et al., 1985). Accordingly, it was hypothesized that HPV would account, in part, for the detrimental effect of hypoxia on aerobic capacity (Naeije, 2011). In support, pharmacological attenuation of HPV in hypoxia using selective vasodilators, such as sitaxsentan, sildenafil, or bosentan, elevates arterial O_2_ saturation (SaO_2_) and improves exercise tolerance, partly restoring aerobic capacity toward normoxic values (Ghofrani et al., 2004; Richalet et al., 2005; Naeije et al., 2010; Olfert et al., 2011; Pham et al., 2012). However, the increase in arterial oxygenation *per se* reported by these studies can also account for the improved exercise performance (Anholm and Foster, 2011).

Nitric oxide (NO), generated from L-arginine by NO synthases, is a vasodilator involved in the regulation of pulmonary vascular tone and therefore HPV (Droma et al., 2002; Scherrer et al., 2013). In normoxia, endothelial NOS plays a key role in the regulation of vascular tone (Ignarro, 1989). However, its activity is suppressed in hypoxic conditions, resulting in decreased NO bioavailability (Bailey et al., 2010a). Such decrease in NO bioavailability is associated with excessive hypoxic pulmonary vasoconstriction, as seen in people susceptible to high-altitude pulmonary edema (Berger et al., 2005). In addition to endothelial NOS, NO can be formed through the nitrate-nitrite-NO pathway. Dietary inorganic nitrate is absorbed by the gastrointestinal system, excreted in the saliva and reduced to nitrite by oral flora (Spiegelhalder et al., 1976). The nitrite is swallowed and transformed to NO in the acid stomach, or used as precursor for NO after intestinal absorption and transport through the circulation elsewhere (Govoni et al., 2008). Hypoxia facilitates the reduction of nitrite to NO (Millar et al., 1998; Maher et al., 2008), allowing more NO to be generated in tissues receiving less O_2_ or that are more metabolically active (Thomas et al., 2001).

Oral and dietary nitrate supplementation has been shown to reduce the O_2_ cost of locomotion (Larsen et al., 2007, 2011; Bailey et al., 2009; Cermak et al., 2012a), which has been attributed to either improved efficiency of oxidative metabolic (Clerc et al., 2007; Larsen et al., 2011) or contractile processes (Bailey et al., 2010b). During exercise in moderate hypoxia, nitrate supplementation improves peripheral O_2_ efficiency and phosphocreatine recovery kinetics (Vanhatalo et al., 2011; Masschelein et al., 2012). Masschelein et al. (2012) showed that nitrate supplementation improved muscle oxygenation during submaximal exercise in severe hypoxia (11% O_2_). These studies assessed performance during incremental exercise (Masschelein et al., 2012) and constant load knee extension exercise (Vanhatalo et al., 2011) to exhaustion, useful exercise paradigms for mechanistic studies, but with limited ecological validity (Currell and Jeukendrup, 2008).

The purpose of this study was two-fold. First, we examined the effect of oral nitrate supplementation on pulmonary hemodynamics at rest and during steady-state submaximal exercise in normoxia and hypoxia. Second, we examined the effect of oral nitrate supplementation on 15 km time trial cycling (TT) performance in normoxia and hypoxia. We tested the following hypothesis: elevating NO bioavailability with nitrate attenuates the development of hypoxic pulmonary hypertension, thereby improving right heart function and exercise performance in severe hypoxia.

## Methods

### Participants

Twelve male cyclists volunteered for this study (age: 31 ± 7 year; weight: 73.5 ± 5.9 kg; maximal aerobic power output: 381 ± 36 W; power-to-weight ratio: 5.2 ± 0.6 W/kg). Inclusion criteria were being a trained cyclist living in Geneva, Switzerland (389–450 m) and a non-smoker, with no previous history of cardiovascular or respiratory disease and not taking any medication. All participants trained at least 3 times/week. All except one were active members of a competitive team and were involved regularly in regional and/or national competitions. All participants gave informed consent prior to participation. The study was approved by the Research Ethics Committee of the University Hospitals of Geneva and conformed to the standards set by the *Declaration of Helsinki*.

### Experimental design

The participants underwent seven experimental testing sessions. Following a full familiarization with the equipment, which included a hypoxia sensitivity test (Bourdillon et al., 2014) and a maximal cardiopulmonary exercise test (incremental cycling to exhaustion) to assess the participant's aerobic fitness (visit one), the participants underwent two experimental phases (placebo and nitrate), each consisting of three exercise sessions (visits two to seven): (1) exercise echocardiography to assess pulmonary hemodynamics in normoxia and hypoxia; (2) 15 km TT in normoxia; and (3) 15 km TT in normobaric hypoxia. TTs were separated by a ~5 day rest period. The placebo/nitrate supplementation was carried out in a double-blind, randomized balanced crossover design, with a 5-day minimum washout period. The TT sessions were conducted in an exercise physiology laboratory, while the steady-state cycling sessions were carried out in an echocardiography room in the university hospital, under similar laboratory conditions (temperature 24.1 ± 1.7°C, humidity 33 ± 5%, barometric pressure 721 ± 6 mmHg). Before each test, the participants refrained from caffeine for 12 h, and heavy exercise and alcohol for 24 h.

#### Oral nitrate supplementation

Before each experimental session, the participants underwent a 3-day oral supplementation with either sodium nitrate (nitrate: 0.1 mmol/kg/day, Larsen et al., 2006) or placebo (sodium chloride, 0.1 mmol/kg/day) in identical gelatin capsules in a double-blinded, randomized order. The participants continued the oral supplementation until all tests were completed within each experimental phase. The participants were instructed to avoid nitrate-rich foods during the entire study period in order to reduce the variations in NO intake during the baseline measurements and thus reduce noise in the collected data. The participants were instructed to fill a qualitative dietary diary throughout the study period to ensure compliance to this dietary restriction.

#### Experimental setup

Throughout each experimental session, the participants wore a nose-clip and breathed through a mouthpiece attached to a low resistance one-way non-rebreathing valve (Hans-Rudolph 2700, Kansas City, KS, USA). The inspiratory side of the valve was connected to a gas mixing system (Altitrainer, SMTec, Nyon, Switzerland) from which they inspired either the normoxic or the hypoxic gas mixture. The fraction of inspired O_2_ (FiO_2_) was kept constant at either 0.21 (normoxia, ambient air) or 0.11 (hypoxia, in Geneva the equivalent of an altitude of ~5000 m). The participants breathed through the same circuit in all six experimental sessions.

### Experimental protocol

#### Echocardiography

The exercise echocardiography sessions comprised 10 min of instrumentation with the participant breathing room-air and seated in semi-supine position (45% angle) on an electronically braked cycle ergometer (Ergoselect 1200, Ergoline GmbH, Bitz, Germany). They were then switched from room air to the gas mixing system for the data collection. After a minimum 4 min adaptation resting measurements were performed after which the participants performed three stages of steady-state submaximal cycling at 50, 100, and 150 W (~6 min each), whilst maintaining a constant pedaling rate of ~70 rpm.

#### Fifteen kilometers time trial cycling

The TT sessions comprised 20 min of instrumentation with the participants breathing room-air while seated on a bicycle fitted to a Computrainer Pro Model trainer (RacerMate®, Seattle, WA, USA). The trainer was calibrated according to the manufacturer's instructions prior to each experimental session. The participants performed a 5-min self-selected warm-up exercise (heart rate < 120 bpm). After return to rest they were then switched to breathe from the gas mixing system for a 4-min post warm-up resting baseline collection. Immediately, following the baseline, the participants performed a 15 km TT as fast as possible. They were free to shift gears and choose pedaling rate. Constant feedback regarding the distance covered, but neither speed nor time, was provided on a computer screen together with a dynamic virtual reality environment showing a cycling avatar (Computrainer™ 3D software version 3.0, Racermate). Two large ventilators were placed ~60 cm in front of the participants and wind velocity was adjusted according to cycling speed.

### Measurements

#### Expired NO concentration

Expired endogenous NO concentration was measured during normoxic rest in duplicate at an expired flow of 50 ml/s by chemoluminescense (FeNO+, Medisoft, Sorinnes, Belgium), once in the placebo and once in the nitrate condition.

#### Echocardiography

Using an echocardiograph (Philips iE33, Andover, MA, USA) and a S5 probe, the following indices of right ventricular systolic function were quantified: (a) systolic right ventricle to right atrium pressure gradient (RV-RA gradient: index of PAP), estimated by peak tricuspid regurgitation velocity (TRV) using the simplified Bernoulli equation (RV-RA gradient = 4TRV^2^); (b) tricuspid annulus plane systolic excursion (TAPSE); and (c) maximal systolic velocity of the tricuspid lateral annulus (S'-wave: index of right heart contractility). For RV-RA gradient measurements, signals were enhanced by venous injection of agitated saline (Lopes et al., 2008). Echocardiography data were analyzed offline using special software (Xcelera, Philips, Andover, MA, USA) by two experienced cardiologists blinded to the conditions. Pulsed O_2_ saturation (SpO_2_) at the middle finger of the right hand was recorded simultaneously to the echocardiography data. These measurements were performed at rest and during the last minute of the steady-state exercise steps.

#### Fifteen kilometers time-trial

##### Performance

Time-to-completion was extracted offline from the Computrainer software.

##### Respiratory variables

Breath-by-breath gas exchange [O_2_ consumption ($\dot{\text{V}}$O_2_) and expired CO_2_ ($\dot{\text{V}}$CO_2_)] and ventilatory flow were measured with a metabolic system (Medgraphics CPX, Loma Linda, CA, USA). Ventilation ($\dot{\text{V}}\text{E}$) and its components tidal volume (VT) and breathing frequency (f) were derived from the flow signal and expressed in l/min BTPS. Partial pressure of end-tidal O_2_ (PetO_2_) and CO_2_ (PetCO_2_) were derived from the expired O_2_ and CO_2_ signals. Prior to each experimental session the system was calibrated using a 3-L syringe (M9474, Medikro Oy, Finland) and precision gas mixtures of known O_2_ and CO_2_ concentrations. The ventilatory equivalent for oxygen ($\dot{\text{V}}\text{E}$/$\dot{\text{V}}$O_2_) ratio was subsequently calculated.

##### Cardiovascular variables

Muscle oxygenation in the left vastus lateralis (~15 cm proximal and 5 cm lateral to the superior border of the patella) was assessed by monitoring changes in oxy- and deoxy-hemoglobin (O_2_Hb and HHb, respectively) obtained with spatially resolved, continuous wave near infrared spectroscopy (NIRS, Artinis Oxymon, MKIII, Zetten, The Netherlands) using a source-detector spacing of 3.8 cm and DPF of 4.0 (Duncan et al., 1995). Muscle total Hb (THb) and delta Hb were calculated using the following equations: THb = O_2_Hb + HHb (Equation 1); delta Hb = O_2_Hb − HHb (Equation 2). Muscle O_2_Hb and HHb were expressed as μmol changes from resting baseline in either normoxia or hypoxia; i.e., resting normoxia for normoxic trials and resting hypoxia for hypoxic trials. This was done because (1) nitrate supplementation potentially alters NIRS baseline as NO is a strong vasodilator and (2) NIRS only gives relative values, and both reasons preclude direct comparison between different baselines. We therefore analyzed the effect of hypoxia and/or nitrate on the magnitude of NIRS signal changes from the resting baseline to the exercise condition.

Beat-to-beat arterial blood pressure (BP) was monitored using finger plethysmography (Finometer® MIDI, Finapress Medical Systems, Amsterdam, The Netherlands). SpO_2_ was measured using earlobe pulse oximetry (Radical-7, Masimo Corporation, Irvine, CA, USA). Heart rate (HR) was derived from the peak-to-peak intervals of the BP signal.

##### Electromyography (EMG)

EMG was recorded from the right vastus lateralis muscle. After shaving, abrading, and degreasing the skin to lower impedance < 5 kΩ, two circular silver-chloride (recording diameter of 10 mm) surface electrodes (Medi-Trace 100, Tyco Healthcare Group, Mansfield, UK) were placed along the line from the superior lateral side of the patella to the anterior superior iliac spine at ~100 mm from the patella with an inter-electrode distance (center-to-center) of 20 mm (2004). Electrode positions were marked on the skin for identical placement between sessions. To minimize movement artifacts electrodes and cables were secured with elastic bandage and netting. The EMG signals were amplified (Bio Amp Powerlab 26T, ADInstruments, Bella Vista, Australia), sampled at 2 kHz, and band pass filtered (10–999 Hz, LabChart version 7.2, ADInstruments, Bella Vista, Australia). The EMG root mean square (RMS) was calculated for each single muscle contraction (LabChart version 7.2, ADInstruments, Bella Vista, Australia).

##### Blood gas variables

Arterialized earlobe capillary blood samples were taken at rest and every 5 km during exercise. Vasodilating cream was applied to an ear lobe 5-min prior to sampling (Decontractyl, Sanofi Aventis, France). The ear lobe was pierced with a lancet and blood collected in 60 μL capillary tubes (MultiCap, Siemens Healthcare Diagnostics Inc., Tarrytown, UK). Samples were analyzed within 5 s using a blood-gas analyzer (Rapidlab 248, Siemens Healthcare Diagnostics Inc., Tarrytown, UK) for arterialized pH (pHa), partial pressures of arterialized O_2_ (PaO_2_) and CO_2_ (PaCO_2_), and arterialized O_2_ saturation (SaO_2_). Standard calibration was performed prior to each experimental session using precision calibration fluids and gas mixtures of known O_2_ and CO_2_ concentrations. Hemoglobin concentration ([Hb]) was measured at rest prior to each exercise session on ear lobe arterialized capillary blood samples using a double wavelength photometer (HemoCue Hb201+, HemoCue AB, Ängelholm, Sweden). CaO_2_ was calculated using the Equation: CaO_2_ = 1.36 × [Hb] × (SaO_2_/100) + 0.003 × PaO_2_ (Equation 3).

With the exception of the blood gas data, which were obtained from the analyzer's print out, all TT data were acquired continuously throughout each experimental session using an analog-to-digital converter (ML880, PowerLab 16/30, ADInstruments, Bella Vista, Australia) with commercially available software (LabChart version 7.2, ADInstruments, Bella Vista, Australia), and stored on a computer for later analysis. The EMG was sampled and recorded at 2 kHz, while all other variables were sampled and recorded at 200 Hz.

### Data analysis and statistics

Resting values were obtained by averaging the data of the last 30 s of the 4 min resting periods prior to the TTs. During the TTs, a mean value for each variable was obtained at each km, by averaging the last 20 s of each km.

One-way ANOVA was used to compare the expired NO concentration between placebo and nitrate conditions at rest in normoxia with an α-level of 0.05 (Matlab R2013b, MathWorks, Natick, MA, USA). Other resting data were analyzed using Two-way repeated measures ANOVA for condition and supplementation effect with an α-level of 0.05. For significant interactions between hypoxia and nitrate, pairwise comparisons were performed using Tuckey's HSD *post-hoc* test with an adjusted α-level of 0.0125. For exercise data, the effects of hypoxia, nitrate, and exercise on mean cardiorespiratory and blood gas responses at rest and during the 15 km TT were assessed using Three-way repeated measures ANOVA with an α-level of 0.05 (Matlab R2013b, MathWorks, Natick, MA, USA). For significant interactions between hypoxia, nitrate, and exercise, pairwise comparisons were performed using Tuckey's HSD *post-hoc* test with an adjusted α-level (in case of interaction between exercise, hypoxia and nitrate an α-level of 0.00078; in case of interaction between exercise and hypoxia or nitrate an α-level of 0.0016; in case of interaction between hypoxia and nitrate an α-level of 0.0125, indicated where appropriate with a superscript ^B^). Additionally to *p*-values, Cohen's d values (effect size) are reported for nitrate or hypoxia effects. Cohen's d was calculated as $d=(M_{1}-M_{2})/\sqrt{(\sigma_{1}^{2}+\sigma_{2}^{2})/2}$, where M_1_ and M_2_ are means of group 1 and 2; σ_1_ and σ_2_ are standard deviations of group 1 and 2. The effect size was considered negligible when *d* < 0.20, small when *d*≥0.20, moderate when *d*≥0.63 and large when *d*≥1.15. Due to missing data, the effects of hypoxia, nitrate, and workload on RV-RA gradient, S'-wave and TAPSE were assessed using linear mixed modeling with an α-level of 0.05 (IBM SPSS Statistics version 22.0; IBM Corporation, Armonk, NY, USA). *Post-hoc* tests were performed on these variables in case of interactions between hypoxia and nitrate using Sidak adjustment for multiple comparisons. Data are reported as means ± SD.

## Results

All 12 subjects completed the entire experimental protocol. No side effects of oral nitrate supplementation were reported.

### Resting variables

#### Expired NO

During normoxic rest, 3-day oral nitrate supplementation increased expired NO by 34 ± 34% compared to placebo (73.5 ± 24.9 ppm vs. 55.5 ± 26.6 ppm, *p* < 0.05, *d* = 0.70, *n* = 11).

#### Respiratory variables

Hypoxia elevated resting VT (*p* = 0.017 vs. normoxia; *d* = 4.17) and tended to lower f (*p* = 0.059), but had no effect on resting $\dot{\text{V}}$E (*p* > 0.05, Table 1). Hypoxia lowered resting PetO_2_, PetCO_2_, SpO_2_, and $\dot{\text{V}}$O_2_ (*p* < 0.05 for all, vs. normoxia), but had no effect on resting $\dot{\text{V}}$CO_2_ (*p* > 0.05, Table 1). Oral nitrate supplementation had no effect on any of the resting respiratory variables (*p* > 0.05 vs. placebo).

**Table 1:** **Effect of hypoxia and oral nitrate supplementation on resting respiratory, cardiovascular, and blood gas variables**.

	**Normoxia**		**Hypoxia**	
	**Placebo**	**Nitrate**	**Placebo**	**Nitrate**
**RESPIRATORY**				
$\dot{\text{V}}$E (L/min)	16.3±4.9	16.3±2.8	17.4±3.9	18.1±3.3
Vt (L)	0.82±0.30	0.81±0.19	1.03±0.41^*^	1.09±0.39^*^
f (br/min)	20.5±4.5	21.2±3.8	18.6±5.2	17.8±5.2
PETCO_2_ (mmHg)	36.8±4.6	37.4±3.1	34.4±3.8^*^	34.1±2.4^*^
PETO_2_ (mmHg)	102.5±6.1	103.7±4.4	43.9±5.4^*^	43.5±4.0^*^
SpO_2_ (%)	97±1	97±1	83±4^*^	84±3^*^
$\dot{\text{V}}$O_2_ (mL/min)	402±44	404±60	339±93^*^	367±65^*^
$\dot{\text{V}}$CO_2_ (mL/min)	450±76	456±64	458±84	480±90
**CARDIOVASCULAR**				
Mean BP (mmHg)	95±14	93±10	89±11	95±10
Systolic BP (mmHg)	125±18	123±13	118±17	128±13
Diastolic BP (mmHg)	75±12	72±8	71±8	76±9
HR (b/min)	73±11	68±15	81±14^*^	82±20^*^
**BLOOD GAS**				
pH	7.47±0.03	7.46±0.04	7.50±0.03^*^	7.47±0.04^*^
PaCO_2_ (mmHg)	31.4±3.7	32.9±2.4	28.4±3.2^*^	29.8±2.3^*^
PaO_2_ (mmHg)	90.0±9.6	92.0±7.3	45.9±4.5^*^	45.0±2.9^*^
SaO_2_ (%)	97±1	97±1	86±5^*^	84±3^*^
[Hb] (g/L)	150±6	151±9	150±8	152±8
CaO_2_ (mL O_2_/L)	203±7	202±13	177±10^*^	174±11^*^
HCO${}_{3}^{-}$ (mmol/l)	20.5±3.3	20.2±3.2	19.5±4.6^*^	19.3±3.9^*^

*Values are mean ± SD*.

* *Different from normoxia (p < 0.05)*.

#### Cardiovascular variables

No changes were observed in resting mean, systolic and diastolic BP with either hypoxia or nitrate (*p* > 0.05). At rest, hypoxia elevated HR (*p* = 0.05, *d* = 3.33, Table 1) and tended to elevate RV-RA gradient (*p* = 0.064, Table 2), while TAPSE tended to be lower (*p* = 0.064 vs. normoxia). Meanwhile, nitrate tended to elevate TAPSE (*p* = 0.081 vs. placebo, Table 2), but had no effect on resting HR or RV-RA gradient (*p* > 0.05). No changes were observed in S'-wave with either hypoxia or nitrate (*p* > 0.05).

**Table 2:** **Effect of hypoxia and oral nitrate supplementation on indices of right heart functions**.

	**Normoxia**		**Hypoxia**	
	**Placebo**	**Nitrate**	**Placebo**	**Nitrate**
**RV-RA GRADIENT**				
Rest	18.6±4.3	20.3±5.5	22.3±7.3^*^	26.1±7.7^*^
50 W	26.0±3.0	27.5±4.51	30.3±6.4^*^	30.0±11.4^*^
100 W	27.3±3.6	29.5±5.1	31.8±8.4^*^	33.7±11.1^*^
150 W	27.5±6.3	29.4±7.6	35.5±6.0^*^	32.1±13.3^*^
**S'-WAVE**				
Rest	12.1±1.6	12.3±1.4	13.0±3.6^*^	12.0±2.1^*^^†^
50 W	14.4±1.1	14.7±1.5	19.1±6.2^*^	15.8±2.7^*^^†^
100 W	15.8±1.3	17.0±1.8	20.2±2.9^*^	18.5±3.2^*^^†^
150 W	19.0±3.7	19.8±1.1	21.7±2.7^*^	20.2±2.8^*^
**TAPSE**				
Rest	20.1±2.8	22.0±2.6	18.8±3.1	20.0±2.7
50 W	24.3±3.3	24.9±4.3	23.9±3.8	25.5±3.4
100 W	28.1±3.4	27.0±4.4	28.2±3.3	27.3±4.2
150 W	30.0±5.9	29.1±4.7	29.0±4.2	30.0±2.6

*Values are mean ± SD. RV-RA gradient (mmHg), systolic right ventricle to right atrium pressure gradient; S'-wave (cm/s), maximal systolic velocity of the tricuspid lateral annulus; TAPSE (mm), tricuspid annular plane systolic excursion*.

* *Different from normoxia (p < 0.05)*.

† *Different from placebo*.

#### Blood variables

Hypoxia lowered resting PaO_2_, PaCO_2_, SaO_2_,and CaO_2_ (*p* < 0.001), and elevated pHa (*p* < 0.05; *d* = 3.97), while [Hb] was unchanged (*p* > 0.05, Table 1). Oral nitrate supplementation had no effect on any of the resting blood variables (*p* > 0.05 vs. placebo).

### Submaximal exercise echocardiography (Table 2)

During steady-state exercise, hypoxia increased the RV-RA gradient (*p* < 0.05 vs. normoxia; *d* = 7.34), while it was unchanged with nitrate (*p* > 0.05 vs. placebo). Hypoxia increased S'-wave during exercise (*p* < 0.001; *d* = 2.55), while nitrate attenuated this rise (interaction: *p* < 0.05). Accordingly, *post-hoc* analysis revealed that the S'-wave was lower with nitrate in hypoxia (*p* < 0.05^B^ vs. placebo hypoxia). No changes were observed in TAPSE with either hypoxia or nitrate (*p* > 0.05). Hypoxia decreased SpO_2_ (*p* < 0.001, *d* = 46.26 vs. normoxia) while it was unchanged with nitrate (*p*>0.001 vs. placebo). Exercise decreased SpO_2_ in hypoxic conditions (*p* < 0.001, for both main effect of exercise and interaction between hypoxia and exercise, Table 2).

### Fifteen kilometers time-trial cycling

#### Performance

Hypoxia increased the time-to-completion by 491 ± 486 s compared to normoxic values (*p* < 0.001; *d* = 2.96). No significant differences were observed in the time-to-completion between placebo and nitrate during TT in normoxia (1581 ± 63 s vs. 1597 ± 96 s, *p* > 0.05) and in hypoxia (2005 ± 309 s vs. 2155 ± 687 s, *p* > 0.05).

#### Respiratory variables (Figure 1)

During TT, hypoxia lowered $\dot{\text{V}}$E by 8 ± 32 l/min during TT (*p* < 0.05 vs. normoxia; *d* = 0.18) while nitrate elevated $\dot{\text{V}}$E by 6 ± 35 l/min in both normoxic and hypoxic conditions (*p* < 0.001 vs. placebo; *d* = 0.24).

**Figure 1:**
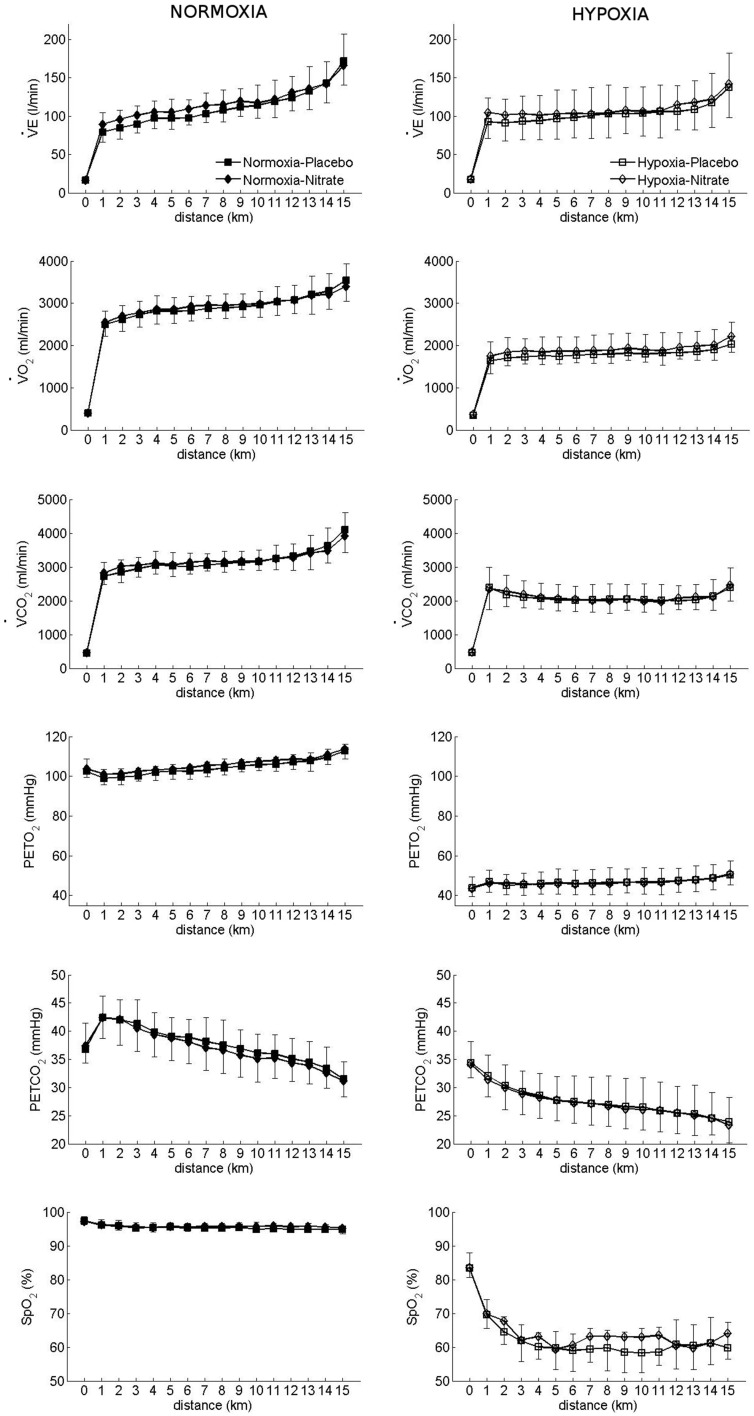
**Effect of hypoxia and nitrate on respiratory variables during 15 km TT**. Filled signs: Placebo, Open signs: nitrate. Mean ± SD.

Hypoxia decreased PetCO_2_ (by 10 ± 4 mmHg) and PetO_2_ (by 59 ± 5 mmHg) during TT (*p* < 0.001 for both, vs. normoxia; *d* = 2.21 and *d* = 9.97, respectively). However, nitrate attenuated this reduction in PetO_2_ with hypoxia (interaction: *p* < 0.05), but had no effect on PetCO_2_ (*p* > 0.05). *Post-hoc* analysis revealed PetO_2_ to be higher during TT in hypoxia with nitrate (*p* < 0.001^B^ vs. placebo hypoxia), while no difference was observed during TT in normoxia with nitrate (*p* > 0.05^B^ vs. placebo normoxia).

Hypoxia decreased both $\dot{\text{V}}$O_2_ and $\dot{\text{V}}$CO_2_ during TT (*p* < 0.001 vs. normoxia; *d* = 2.71 and *d* = 2.37, respectively, Figure 1), while nitrate increased $\dot{\text{V}}$O_2_ (*p* < 0.05 vs. placebo; *d* = 0.14) but had no effect on $\dot{\text{V}}$CO_2_ (*p* > 0.05).

Hypoxia increased $\dot{\text{V}}$E/$\dot{\text{V}}$O_2_ during TT (38.4 ± 7.6 l/lO_2_ vs. 55.8 ± 13.1 l/lO_2_, *p* < 0.001, *d* = 1.63), while nitrate enhanced it in normoxia but not in hypoxia (interaction: *p* < 0.05). Accordingly, $\dot{\text{V}}$E/$\dot{\text{V}}$O_2_ was higher with nitrate in normoxia (37.4 ± 8.2 l/lO_2_ vs. 39.6 ± 7.3 l/lO_2_, nitrate vs. placebo, *p* < 0.05), but it was not different in hypoxia (56.5 ± 13.8 l/lO_2_ vs. 54.3 ± 12.2 l/lO_2_, *p* > 0.05).

#### Cardiovascular variables

Hypoxia decreased mean BP during 15 km TT (*p* < 0.001 vs. normoxia; *d* = 0.54), while nitrate attenuated this decrease (interaction: *p* < 0.001). Post-hoc analysis showed that hypoxia decreased mean BP by 13 ± 14 mmHg with placebo (*p* < 0.001^B^ vs. placebo normoxia), while nitrate increased BP in hypoxic conditions by 6 ± 15 mmHg (*p* < 0.001^B^ vs. placebo hypoxia). No change was observed in mean BP during TT with nitrate in normoxia (*p* > 0.05 vs. normoxic placebo). Hypoxia increased HR during TT by 10 ± 12 bpm (*p* < 0.001 vs. normoxia; *d* = 0.42), while no change was observed with nitrate (*p* > 0.05). During TT, hypoxia lowered SpO_2_ (*p* < 0.001 vs. normoxia; *d* = 5.09), while nitrate blunted this decrease (interaction: *p* = 0.018). Specifically, SpO_2_ was lower with hypoxia by 33 ± 8% in placebo (*p* < 0.001^B^ vs. placebo normoxia) and by a lesser extent (31 ± 9%) in nitrate (*p* < 0.001^B^ vs. placebo normoxia, Figure 1).

#### Muscular variables (Figure 2)

There was an interaction between hypoxia and nitrate during TT on muscle O_2_Hb (interaction: *p* < 0.05). As a result O_2_Hb was lower in the nitrate hypoxia condition compared to nitrate normoxia. Hypoxia lowered muscle delta Hb by 12 ± 14 μM (*p* < 0.001 vs. normoxia; *d* = 0.56) and tended to lower muscle THb during TT (*p* = 0.053 vs. normoxia). Meanwhile, there was a tendency for muscle delta Hb to be elevated with nitrate (*p* = 0.061 vs. placebo), while no difference was observed in muscle THb (*p* > 0.05 vs. placebo). No differences were observed in muscle HHb during TT with either hypoxia or nitrate (*p* > 0.05). Hypoxia decreased muscle EMG RMS during TT by 62 ± 64 μV (*p* < 0.001 vs. normoxia), while no changes were observed with nitrate (*p* > 0.05 vs. placebo).

**Figure 2:**
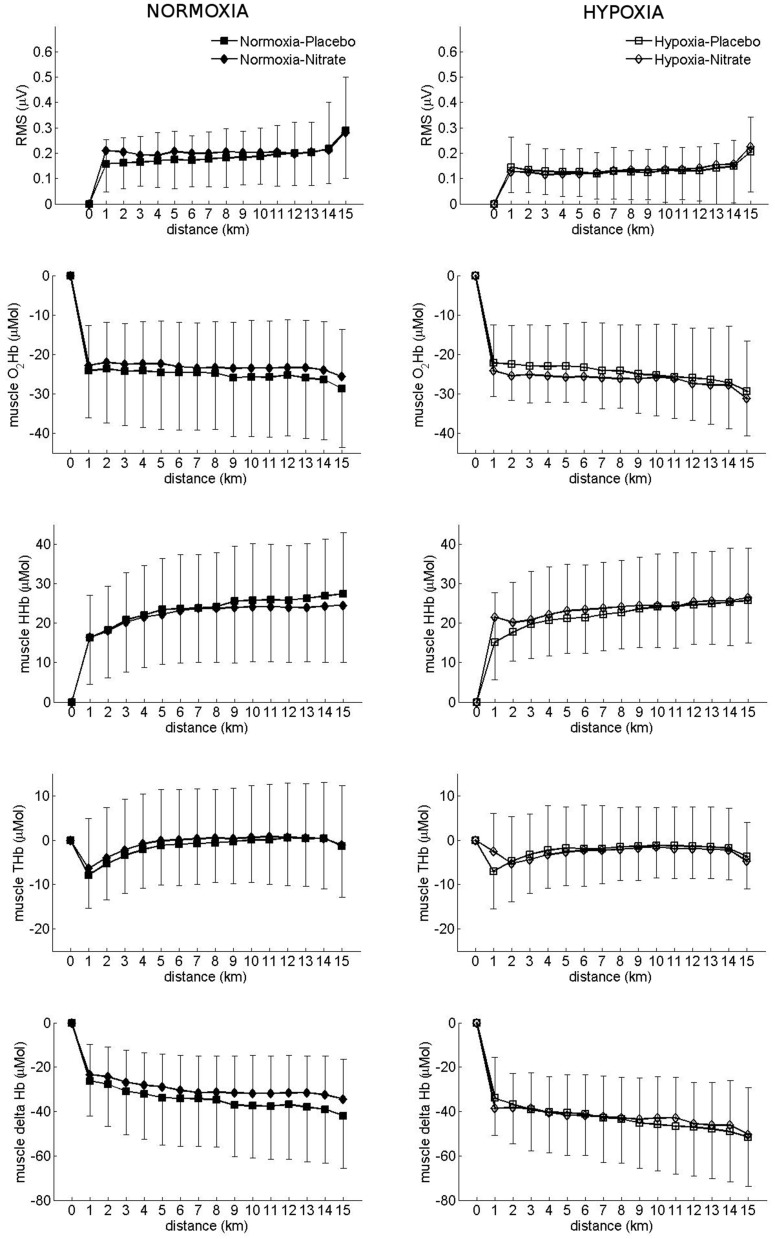
**Effect of hypoxia and nitrate on muscular variables during 15 km TT**. Filled signs: Placebo, Open signs: nitrate. Mean ± SD.

#### Arterialized blood gas variables (Table 3)

Hypoxia increased pH during TT (*p* < 0.001 vs. normoxia), while nitrate had no effect (*p* > 0.05 vs. placebo). Hypoxia decreased PaCO_2_, PaO_2_, and SaO_2_ during TT (*p* < 0.001 vs. normoxia; *d* = 1.29, *d* = 5.83, *d* = 3.77, respectively), while no changes were observed with nitrate (*p* > 0.05 vs. placebo). Similarly, HCO${}_{3}^{-}$ concentration was lowered during TT in hypoxia (*p* = 0.013 vs. normoxia, *d* = 0.26), while nitrate had no effect (*p* > 0.05 vs. placebo).

**Table 3:** **Effect of hypoxia, nitrate supplementation and exercise on arterialized blood samples**.

	**Normoxia**		**Hypoxia**	
	**CON**	**NO**	**CON**	**NO**
**pH**				
Rest	7.47±0.03	7.47±0.04	7.50±0.03^*^	7.47±0.04^*^
Km 5	7.41±0.05^#^	7.39±0.04^#^	7.44±0.07^#^^*^	7.44±0.05^#^^*^
Km 10	7.40±0.04^#^	7.39±0.05^#^	7.42±0.09^#^^*^	7.43±0.06^#^^*^
Km 15	7.37±0.05^##^	7.38±0.04^##^	7.42±0.09^#^^*^	7.40±0.06^##^^*^
**PaO**_2_				
Rest	91.9±7.3	92.9±7.0	46.2±4.5^*^	44.7±2.9^*^
Km 5	78.8±5.1	80.3±8.8	32.8±1.5^#^^*^	34.7±4.7^#^^*^
Km 10	79.3±7.9^##^	81.5±6.7^##^	32.9±2.9^#^^*^	33.8±3.7^#^^*^
Km 15	77.6±7.5^##^	78.9±7.8^##^	33.4±2.4^##^^*^	33.3±1.5^##^^*^
**PaCO**_2_				
Rest	30.9±3.4	32.8±2.4	28.1±3.1^*^	29.4±1.9^*^
Km 5	30.8±3.0^#^	29.4±2.6^#^	21.9±3.7^#^^*^	22.8±2.6^#^^*^
Km 10	27.5±2.4^#^	26.9±1.9^#^	19.5±3.1^#^^*^	20.6±2.7^#^^*^
Km 15	24.6±2.6^#^	24.8±1.6^#^	18.2±2.7^#^^*^	18.5±2.9^#^^*^
**SaO**_2_				
Rest	97.4±0.6	97.3±0.8	86.2±4.7^*^	83.8±3.0^*^
Km 5	95.8±0.8	95.8±1.2	67.1±3.9^#^^*^	69.1±6.8^#^^*^
Km 10	95.7±1.0	96.0±0.7	65.6±4.2^#^^*^	68.0±5.0^#^^*^
Km 15	95.1±1.1	95.5±1.2	66.8±2.4^#^^*^	66.3±3.8^#^^*^
**HCO**_3_				
Rest	23.8±0.9	24.8±2.1	24.1±1.2^*^	22.8±1.8^*^
km 5	20.9±3.0^#^	19.7±1.9^#^	18.3±3.9^#^^*^	19.0±2.8^#^^*^
km 10	19.3±2.2^##^	18.6±2.0^##^	16.5±3.9^##^^*^	17.9±4.2^##^^*^
km 15	17.0±2.4^##^	17.6±1.7^##^	16.4±3.8^##^^*^	16.1±2.5^##^^*^

*Values are mean ± SD. PaO_2_, arterialized O_2_ pressure (mmHg); PaCO_2_, arterialized CO_2_ pressure (mmHg); SaO_2_, arterialized O_2_ saturation (%); HCO${}_{3}^{-}$, bicarbonates concentration (mmol/l)*.

* *Different from normoxia (p < 0.05)*.

# *Different from rest*.

## *Different from km 5, (p < 0.05 for both)*.

## Discussion

We examined the effects of oral nitrate supplementation on pulmonary arterial pressure, time trial performance, and muscle tissue oxygenation, during exercise in normoxia and hypoxia in a group of trained cyclists. We found that, despite increased expired NO, which suggested increased NO bioavailability, no major improvements in right heart functions nor TT performance were observed with oral nitrate supplementation during exercise in normoxia and hypoxia. We found evidence of greater muscle tissue deoxygenation during exercise in hypoxia with nitrate supplementation. Nevertheless, these changes failed to affect 15 km TT performance. Our findings indicate that oral nitrate supplementation does not attenuate the development of acute hypoxia-induced pulmonary hypertension or improve TT performance in normoxia and acute hypoxia in well-trained cyclists.

### Hypoxic pulmonary vasoconstriction

NO inhalation is known to reverse HPV (Frostell et al., 1993; Scherrer et al., 1996), while intravenous sodium nitrite infusion (1 μmol/min) has been shown to blunt the hypoxia-induced increase in pulmonary arterial systolic pressure by ~25% during 3 h exposure to moderate hypoxia (Kelly et al., 2014). In the present study, we used the RV-RA gradient as a surrogate measure of systolic pulmonary artery pressure and found it to be elevated during steady-state exercise in hypoxia compared to normoxic exercise (Table 2), which is indicative of HPV. Contrary to our hypothesis, oral nitrate supplementation failed to attenuate the RV-RA gradient at rest or during steady-state submaximal exercise in acute hypoxia (Table 2). However, it should be acknowledged that HPV has several temporal components (Swenson, 2013). The second phase of the HPV response, which contributes around two-third of the increase in systolic pulmonary arterial pressure, only plateaus after 2 h of exposure to hypoxia (Talbot et al., 2005). In the present study, we assessed right heart function during acute hypoxic exposure (~30 min) and therefore cannot exclude effects after a 1–2 h exposure. Therefore, it is possible that the lack of major improvements in RV-RA gradient in our study could be due to the acute nature of our hypoxic stimulus. Alternatively, it is possible that nitrate-nitrite-NO levels in the lungs were too low for a significant effect on the pulmonary vasculature, even though the large increase in expired NO would suggest the contrary.

### Performance

Numerous studies examined the effect of acute or chronic nitrate supplementation on TT performances in normoxia, and found either improvement (e.g., Naeije et al., 2010; Masschelein et al., 2012; Hoon et al., 2014) or no change in the time-to-completion (Cermak et al., 2012b; Peacock et al., 2012; Christensen et al., 2013; Boorsma et al., 2014; Glaister et al., 2014; Hoon et al., 2014; Lane et al., 2014). A meta-analysis of 17 studies by Hoon et al. (2013) revealed a small, but insignificant beneficial effect of nitrate supplementation on normoxic TT performance. In trained cyclists, Cermak et al. (2012a) reported a ~10 s improvement in 10 km TT performance in normoxia following a 6-day beetroot juice supplementation, while Muggeridge et al. (2014) observed a 38 s improvement in 16.1 km TT performance in moderate hypoxia (simulated 2500 m). In agreement with these previous findings, we observed non-significant improvements in 15 km TT performance during normoxia (by ~16 s) and severe hypoxia (by ~151 s), following 3-day oral nitrate supplementation in a group of trained cyclists. Taken together, it would appear that nitrate supplementation has only marginal effects on time trial performance in well-trained athletes.

### Muscle haemodynamics

Larsen et al. (2007) demonstrated an O_2_-sparing effect of oral nitrate supplementation. They reported reduced $\dot{\text{V}}$O_2_ during steady-state submaximal cycling following a 3-day nitrate supplementation compared to placebo. In addition to liberating bioactive NO, plasma nitrite by itself acts as both a potent vasodilator in hypoxia (Maher et al., 2008) and as an alternative electron acceptor, replacing O_2_ in respiration (Basu et al., 2008) and thus partly accounting for the O_2_ sparing effect of dietary nitrate during exercise. Accordingly, we would expect such “O_2_-sparing effect” of nitrate supplementation to be reflected in muscle tissue oxygenation, $\dot{\text{V}}$O_2_ and performance, particularly under hypoxic conditions.

In normoxia, Glaister et al. (2014) found no effect of nitrate supplementation on power output, muscle tissue oxygenation, performance, or integrated EMG activity during 20 km TT. Likewise, Masschelein et al. (2012) reported no difference in muscle O_2_Hb between a 6-day nitrate supplementation and control during steady-state cycling in hypoxia compared to control. However, they observed an improvement in muscle tissue oxygenation index and reduced muscle HHb during hypoxic exercise with nitrate, which was attributed to higher $\dot{\text{V}}$E and SpO_2_ associated with a respiratory effect of increased NO (see “*Respiratory effects of nitric oxide”*). Meanwhile, Vanhatalo et al. (2011) found improved phosphocreatine recovery kinetics with nitrate supplementation, which reduced substrate utilization and attenuated fatigue development during high intensity knee extension in moderate hypoxia, restoring high intensity isometric exercise tolerance of short duration (~24 s) to normoxic values. From these observations, they concluded that dietary nitrate improves skeletal muscle energetics and functional capacity during exercise in moderate hypoxia. In contrast to these previous findings, we found a reduction in muscle O_2_Hb during the 15 km TT with oral nitrate supplementation, while EMG RMS was unchanged (Figure 2). As performance was not significantly different with nitrate supplementation, we attribute the greater reduction in muscle tissue oxygenation to a better matching of O_2_ delivery to the metabolic requirements of the working muscles (Thomas et al., 2001).

### Respiratory effects of nitric oxide

Unexpectedly, we found higher $\dot{\text{V}}$E during exercise in both normoxia and hypoxia with nitrate supplementation, accompanied by higher SpO_2_ during exercise in hypoxia. Our findings confirm previous reports of increased SpO_2_ (by 1.5–3.5%) with nitrate supplementation during TT in normoxia and also steady state submaximal cycling in hypoxia (Masschelein et al., 2012; Puype et al., 2015). However, in contrast to our data, Masschelein et al. (2012) did not find any changes in $\dot{\text{V}}$E during hypoxic exercise with nitrate, while Puype et al. (2015) did not report any $\dot{\text{V}}$E values. At the level of the peripheral chemoreceptors nNOS-catalyzed production of NO is thought to be an O_2_-dependent process, with NO exerting an inhibitory influence on the carotid body activity (Prabhakar and Semenza, 2012), a mechanism difficult to reconcile with our observation of increased $\dot{\text{V}}$E with oral nitrate supplementation. The possible role of nitrate supplementation in non-NOS dependent formation of NO and ventilation during exercise warrants further investigation.

### Responders vs. non-responders?

Alternatively, a potential explanation for the lack of improvement in performance could be the existence of responders and non-responders to oral nitrate supplementation, perhaps in line with differences in training status. In support, data from Bescós et al. (2012) showed that 7 out of 13 trained athletes were non-responders, with only small elevations in plasma nitrate concentration observed following a 3-day nitrate supplementation. Similarly, Boorsma et al. (2014) reported that only 2 of their 8 runners improved 1500 m running performance following an 8-day nitrate supplementation. Likewise, 8 out of 12 of our participants improved their normoxic 15 km TT performance with oral nitrate supplementation, while 7 participants improved their times with nitrate supplementation in hypoxia. Trained athletes have higher plasma nitrate and nitrite concentrations, and express more muscle nNOS protein compared to untrained controls (Poveda et al., 1997; McConell et al., 2007). These findings led, Wilkerson et al. (2012) to speculate that the elevated plasma nitrate and nitrite concentrations seen in highly trained athletes, would lower the impact of oral nitrate on NO bioavailability in these individuals. They further speculated that increased muscle nNOS activity could lessen the importance of the nitrate-nitrite-NO pathway on NO production. Recently, Porcelli et al. (2014) observed a greater reduction in the O_2_ cost of exercise and an improvement in 3-km performance in individuals with low aerobic fitness compared to those with high aerobic fitness following a 6-day nitrate supplementation (5.5 mmol/day). The authors concluded that the ergogenic effects of nitrate supplementation depend on the individual's aerobic fitness level and are related to the relative changes in plasma nitrate and nitrite concentrations. Since the participants in our study were well-trained athletes it is therefore possible that their training status and associated high muscle nNOS activity might have, in part, attenuated any effects of oral nitrate supplementation on TT performance.

### Methodological considerations

An important methodological consideration when interpreting our findings is the lack of plasma nitrate and nitrite measurements in our study. In recent years, numerous studies have assessed the effects of acute and chronic nitrate supplementation using sodium nitrate (~0.1–0.3 mmol/kg/day Larsen et al., 2006; Bescós et al., 2012) or beetroot juice (~5.5–9.6 mmol/day, Bailey et al., 2009; Lansley et al., 2011; Vanhatalo et al., 2011; Bond et al., 2012; Cermak et al., 2012b; Fulford et al., 2013; Kelly et al., 2013, 2014), and reported increases in plasma nitrate (~500%) and nitrite (~40–140%), regardless of supplementation duration (i.e., from a single dose to 15 days of supplementation). In the present study, we observed a 30% increase in expired NO, a measure of NO bioavailability at the level of the lung, following oral nitrate supplementation (0.1 mmol/kg/day, i.e., an average individual dose ~7.3 mmol/day). Since the oral nitrate supplementation regimen used in the present study was comparable those studies which were successful in elevating plasma levels of nitrate and nitrite, we contend that nitrate and nitrite bioavailability was likely to be elevated in our subjects.

Another critique is the use of systolic RV-RA gradient as a surrogate index for PAP in the present study. Since changes in the RV-RA gradient are influenced directly by changes in cardiac output, and are subject to measurement errors (Fisher et al., 2009), they do not necessarily reflect changes in PVR. Nevertheless, our work is the first to combine the study of the effect of nitrate supplementation on performance and on right heart function. The next step would be to invasively measure PAP using catheterization, which is ethically questionable in healthy well-trained athletes, especially given the expected limited beneficial effects of nitrate.

We used ear-lobe arterialized blood which does not necessarily faithfully reflect arterial blood saturation, specifically during heavy exercise when sampling is difficult. The arterial-end-tidal oxygen and CO_2_ differences suggest that some contamination with ambient air occurred. However, our interpretations of results would not radically change if PaO_2_ and PaCO_2_ were slightly over-, respectively underestimated.

## Conclusions

We found that 3-day oral nitrate supplementation elevated expired NO but did not improve right heart function during hypoxic exercise. Despite increased NO bioavailability we did not observe an improvement in 15 km time trial performance in normoxia and severe hypoxia with nitrate supplementation. Our findings do not support nitrate supplementation in preventing the development of hypoxia-induced pulmonary hypertension and improving exercise performance in hypoxia in trained athletes.

## Author contributions

BK and PM contributed to the conception and design of the experiment. JF, NB, PM, and BU performed the data collection. PM and HM led the analysis of the echocardiography data, while NB and JF led the analysis of the TT data. NB led the interpretation of the data and both NB and JF drafted the manuscript. NB prepared the figures. PM, BU, and BK contributed in the revision of the manuscript. NB and JF contributed equally and share first authorship. All authors approved the final version of the manuscript.

### Conflict of interest statement

The authors declare that the research was conducted in the absence of any commercial or financial relationships that could be construed as a potential conflict of interest.
